# miR-451a abrogates treatment resistance in FLT3-ITD-positive acute myeloid leukemia

**DOI:** 10.1038/s41408-018-0070-y

**Published:** 2018-03-21

**Authors:** Rosanna H. E. Krakowsky, Alexander A. Wurm, Dennis Gerloff, Christiane Katzerke, Daniela Bräuer-Hartmann, Jens-Uwe Hartmann, Franziska Wilke, Christian Thiede, Carsten Müller-Tidow, Dietger Niederwieser, Gerhard Behre

**Affiliations:** 1 Division of Hematology and Oncology, University Hospital Leipzig, Leipzig, Germany; 2 Department of Dermatology and Venereology, University Hospital Halle, Halle (Saale), Germany; 3 Medical Clinic and Polyclinic 1, Carl Gustav Carus University Hospital at the Technical University Dresden, Dresden, Germany; 4 Department of Medicine V, Hematology, Oncology and Rheumatology, University Hospital of Heidelberg, Heidelberg, Germany

Acute myeloid leukemia (AML) is a fatal disease with up to 95% of patients remaining incurable. While supportive care has been increased in quantity and quality; substantial progress of AML therapy itself is still lacking. Primarily two predicaments challenge current therapy: first, the heterogeneity of the disease; second, the phenomenon of treatment resistance. Although many AML patients initially respond to therapy, the majority of patients relapse caused by chemoresistant clones, ultimately leading to the incurability of the disease^1–3^.

Approximately a quarter of AML patients present with FLT3-gain-of-function mutations. This usually entails a constitutive activation of FLT3 receptor downstream signaling pathways and changes gene expression patterns from healthy to malignant^4^. On account of the poor prognosis of FLT3-ITD^+^ AML patients, FLT3-inhibitors are under development and in clinical testing. Nevertheless, acquired treatment resistance persists. This underlines the necessity of a greater knowledge on the origins of resistance as well as new approaches abrogating treatment resistance^5^.

Therefore, we propose to deviate from the common strategy of target inhibition and focus on the reinforcement of negatively regulated downstream targets of well-known oncogenes such as FLT3-ITD.

Because microRNA (miR) expression was shown to be altered by FLT3-ITD, this group of small non-coding RNAs is of interest in this work. By binding to the 3’UTRs of mRs, miRs post-transcriptionally regulate gene expression. Thus, miRs hold key regulatory functions in processes such as hematopoiesis and leukemogenesis^6–8^. In accordance with this, various miRs have been shown to impact treatment resistance as well as sensitivity toward AML therapy^9^. First clinical trials disclosed the potential of miR-inhibitors to be a promising therapeutic option^10^. Nevertheless, no miR-based experimental drugs are currently available in AML.

Therefore, we screened for differentially expressed miRs in bone marrow mononuclear cells from healthy donors and AML patients with either FLT3-wildtype, mutations in the FLT3-tyrosine kinase domain (TKD) or FLT3-ITD mutations (Fig. 1a, Supplementary Table 1). Here, we found miR-451a to be significantly (*p* = 0.002) lower expressed in FLT3-ITD^+^ AML patients compared to healthy individuals; while among the remaining AML samples screened, miR-451a levels were lowest in FLT3-ITD patient samples (Fig. 1b). In agreement with this, we observed increasing miR-451a levels upon differentiation of human and murine hematopoietic cells (Supplementary Figure 1a-c). In addition, we analyzed the AML patient cohort of “The Cancer Genome Atlas” (TCGA)^11^ and discovered a similar miR-451a expression distribution (Fig. 1c). In order to confirm our observations in vitro, we assessed miR-451a levels by qPCR in FLT3-ITD-transduced U937 and 32D cells (Fig. 1d,e). Indeed, a stable overexpression of FLT3-ITD led to a decrease in miR-451a levels.

**Fig. 1:**
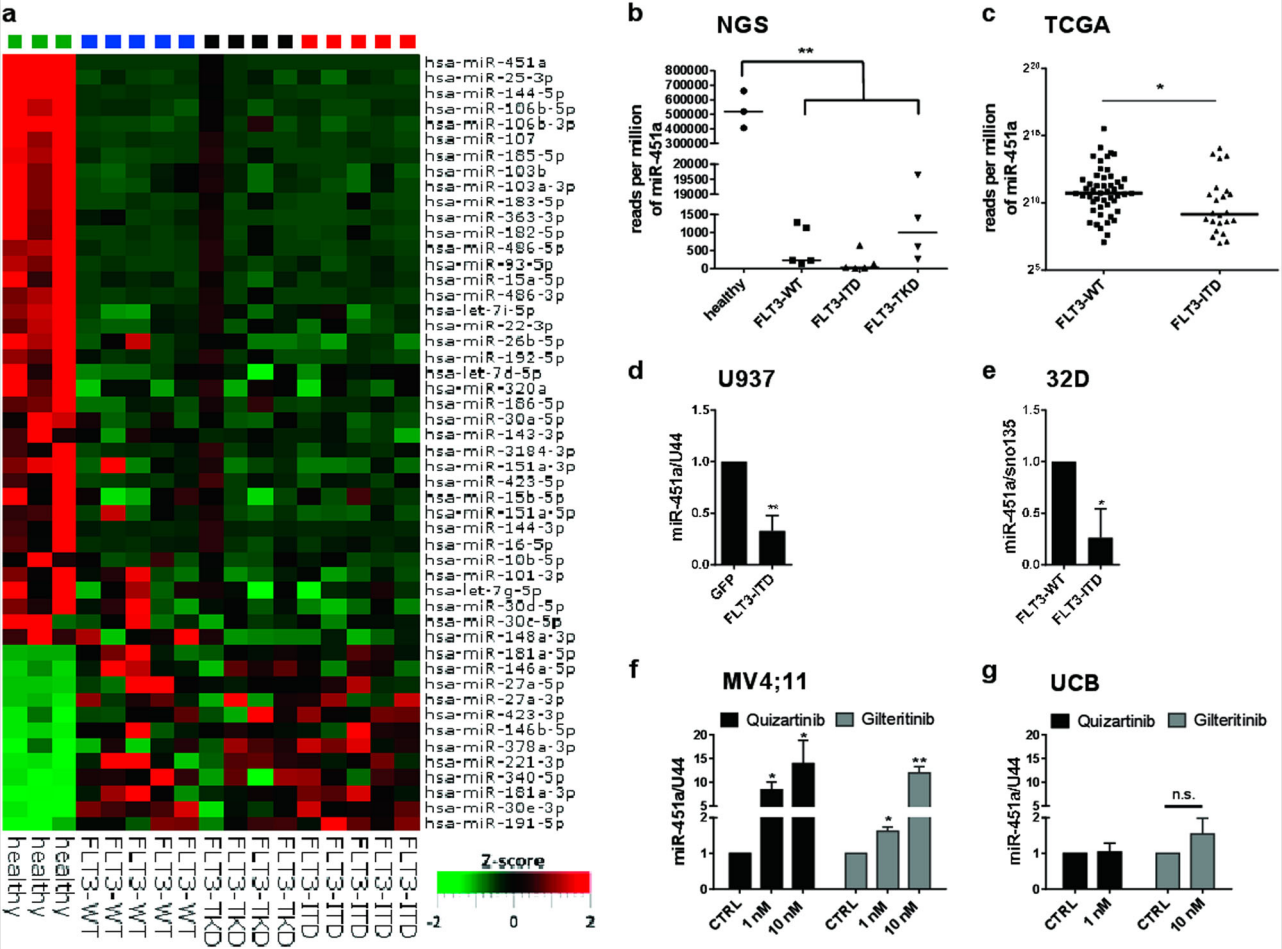
FLT3-ITD downregulates miR-451a. **a** NGS of bone marrow mononuclear cells from healthy donors and normal karyotype AML patients with FLT3-WT, FLT3-ITD or FLT3-TKD. Heatmap of the top 50 miRs differentially expressed in AML samples compared to healthy donors ranked by *p*-value. miR-451a exhibited the lowest *p*-value. **b** Median expression levels of miR-451a are lowest in FLT3-ITD^+^ AML samples. Significance was determined by Wilcoxon test (two-tailed **p < 0.01). **c** MiR-451a expression among FLT3-WT and FLT3-ITD AML patients of the TCGA cohort. Patients depicted were of normal karyotype. Significance was determined by Wilcoxon test (two-tailed *p < 0.05). **d**,**e** Transduction of U937 and 32D cells with FLT3-ITD led to significant decrease of miR-451a levels, measured by qPCR. (two-tailed *t*-test *p < 0.05; **p < 0.01). **f** MiR-451a levels of MV4;11 cells after 48 h treatment with FLT3 inhibitors quizartinib (black bars) and gilteritinib (gray bars). Both FLT3 inhibitors raised significantly miR-451a levels measured by qPCR. (two-tailed *t*-test *p < 0.05; **p < 0.01). **g** MiR-451a expression in umbilical cord blood (UCB) cells does not change upon 48 h treatment with quizartinib and gilteritinib. (two-tailed *t*-test; n.s. *p* > 0.05). All experiments were performed at least three times

In contrast, interruption of FLT3-ITD signaling by treatment of MV4;11 cells with FLT3 inhibitors quizartinib and gilteritinib (currently evaluated in clinical trials) caused an increase in miR-451a expression (Fig. 1f). As a healthy control, we treated mononuclear cells from umbilical cord blood (UCB) with quizartinib as well as gilteritinib and observed neither a significant change in miR-451a expression nor a difference in cell viability compared to the control (Fig. 1g, Supplementary Figure 2a). In addition, we demonstrated that only cells carrying FLT3-ITD were targeted by the indicated inhibitors (Supplementary Figure 1b-d, f).

Since miR-451a correlates with an increased cancer persistence as well as recurrence^12^, we focused on the possible underlying interaction axis.

In various cancer types it was shown that mRNA of multi drug resistance protein 1 (MDR1), an ATP dependent efflux pump mediating chemoresistance, is targeted by miR-451a^12^. Presently, nothing is known about the FLT3-ITD-miR-451a-MDR1 axis in AML. Hence, we conjectured that FLT3-ITD-induced repression of miR-451a and subsequent increase in MDR1 protein is in part responsible for the poor therapy response of FLT3-ITD^+^ AML patients. To prove this, we measured MDR1 protein amount after exogenous FLT3-ITD introduction and observed elevated MDR1 levels in FLT3-ITD transduced U937 cells by flow cytometry (Fig. 2a) and western blot (Fig. 2b). Upon treatment with quizartinib and gilteritinib, both miR-451a increment and decrease in MDR1 protein levels were detected (Fig. 2c).

**Fig. 2:**
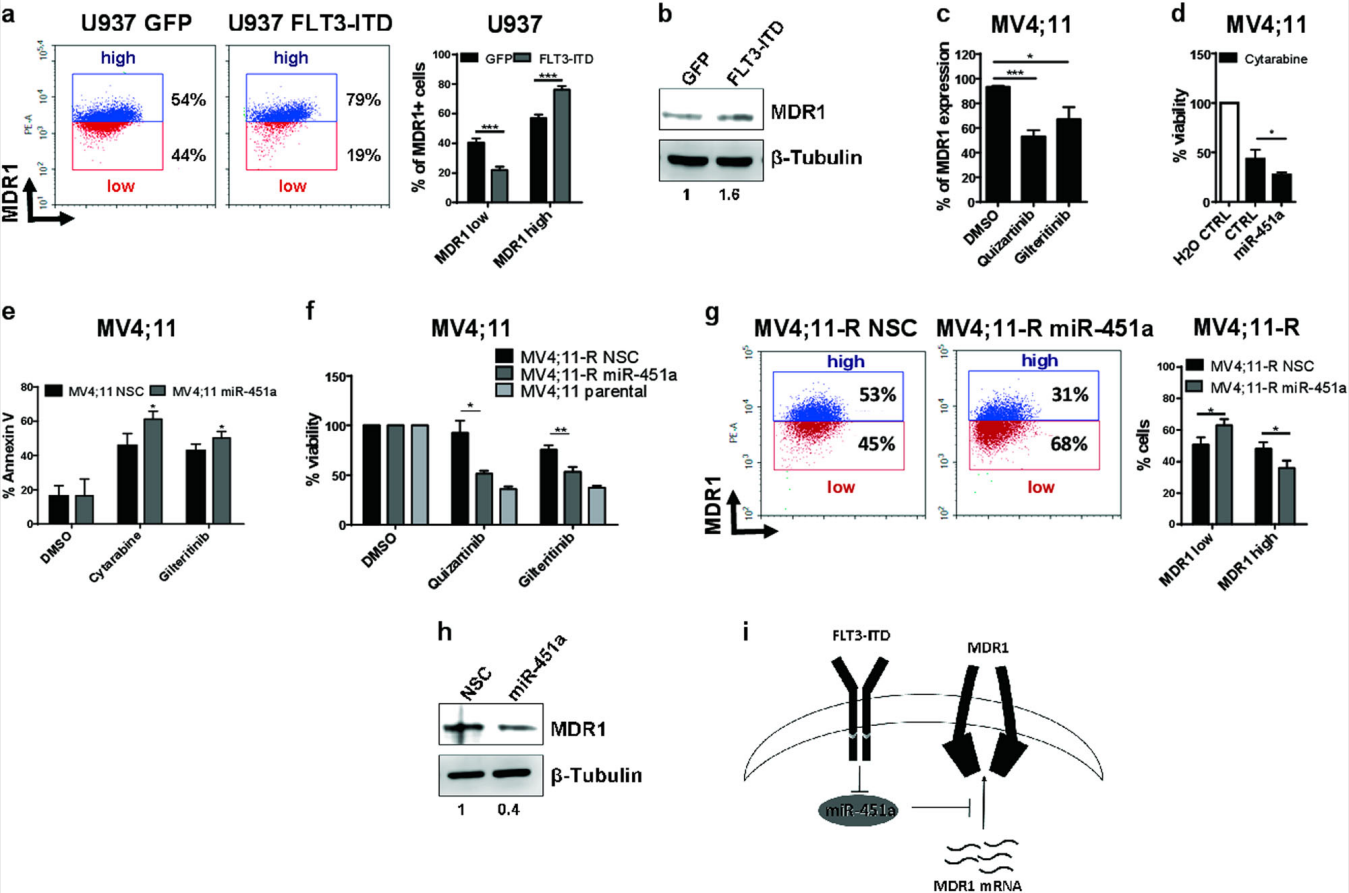
MiR-451a sensitizes FLT3-ITD^+^ cells to chemotherapy. **a** Stable transduction of U937 cells with FLT3-ITD and GFP. In comparison to the GFP control, transduced FLT3-ITD led to increased MDR1 levels in U937 cells (two-tailed *t*-test ***p < 0.001). **b** Western blot analysis revealed increased MDR1 protein levels in FLT3-ITD transduced U937 cells in comparison to GFP control (**c**) MV4;11 cells treated for 48 h with quizartinib and gilteritinib. Flow cytometric analysis exhibited decreased MDR1 protein levels (two-tailed *t*-test *p < 0.05; **p < 0.01). **d** Transient transfection of miR-451a resulted in enhanced sensitivity of MV4;11 cells toward cytarabine. Cell viability was measured by MTS-assay (two-tailed *t*-test *p < 0.05). **e** Annexin V levels of stable miR-451a-transduced MV4;11 parental cells after 48 h treatment. Flow cytometry revealed a significant increment of Annexin V^+^ cells in miR-451a transduced cells compared to the non-silencing control (NSC) (two-tailed *t*-test *p < 0.05). **f** MiR-451a abrogates treatment resistance of MV4;11-R cells. Resistant MV4;11 cells were stably transduced with miR-451a and treated for 48 h with quizartinib or gilteritinib. NSC non-silencing control (two-tailed *t*-test *p < 0.05; **p < 0.01). **g** MV4;11-R cells transduced with miR-451a showed decreased MDR1 levels compared to the NSC. All experiments were performed at least three times (two-tailed* t*-test *p < 0.05). **h** Western blot analysis revealed reduced MDR1 protein levels in miR-451a transduced MV4;11-R cells in comparison to NSC control. **i** Proposed scheme of the FLT3-ITD-MDR1-miR-451a axis

To corroborate our hypothesis, we determined miR-451a levels in various cell lines and measured corresponding MDR1 expression. Here, expression of MDR1 correlated inversely with miR-451a levels in the leukemic cell lines K562, MV4;11, and U937 (Supplementary Figure 3a, b). Next, stable transduction of miR-451a in MV4;11 cells resulted in a significant decrease of MDR1 protein levels compared to the control (Supplementary Figure 3c). Furthermore, luciferase assay with the 3’UTR of MDR1 and mutated MDR1 3’UTR in MV4;11 cells verified that MDR1 is a direct target of miR-451a in the AML background (Supplementary Figure 3d).

To investigate whether miR-451a alone is sufficient to increase chemosensitivity, we transiently transfected MV4;11 cells with miR-451a followed by a treatment with cytarabine for 48 h. Via MTS assay, we detected a significant reduction of cell viability (Fig. 2d). By means of Annexin V staining and flow cytometry, we determined a significant additive effect on the percentage of apoptotic cells upon cytarabine as well as gilteritinib treatment in combination with exogenous miR-451a expression (Fig. 2e).

In pursuance of the miR-451a-induced abrogation of chemoresistance, we included the treatment-resistant subclone MV4;11-R into our research. MV4;11-R was previously established by Stölzel et al. by continuous midostaurin treatment of parental MV4;11 cells^13^. MV4;11-R cells were shown to lack histone methyltransferase *EZH2* correlating with decreased sensitivity toward multiple drugs including cytarabine and quizartinib^14^. In addition, we found an increased resistance toward gilteritinib in these cells (Supplementary Figure 2e). Upon treatment with either quizartinib, gilteritinib or cytarabine, cell growth was inhibited in MV4;11 parental cells, whereas MV4;11-R cells only showed decelerated growth (Supplementary Figure 2f-h). Consequently, we stably transduced MV4;11-R cells with miR-451a and observed a restored drug sensitivity toward FLT3 inhibitors quizartinib and gilteritinib (Fig. 2f). Moreover, exogenous miR-451a resulted in decreased MDR1 protein levels as measured by flow cytometry (Fig. 2g) and western blot (Fig. 2h).

As proof of principle, we were interested whether MDR1 inhibitor tariquidar was able to mimick miR-451a-mediated effects on chemoresistance toward quizartinib. Therefore, we treated MV4;11 as well as MV4;11-R cells with tariquidar in combination with quizartinib and analyzed cell viability. Here, tariquidar enhanced quizartinib-mediated effects and partially abrogated chemoresistance of MV4;11-R cells (Supplementary Figure 3e).

Overall, our data give insights into one molecular cause of the poor prognosis conferred by FLT3-ITD. We demonstrated that MDR1 expression is indirectly targeted by FLT3-ITD through miR-451a (Fig. 2i). Current treatment drafts of FLT3-ITD^+^ AML patients trend toward inhibition of FLT3-ITD function; since these encounter resistance impediments similar to the standard treatment regimen, sole inhibition appears to be insufficient. Hence, we propose to not only focus on inhibition of FLT3-ITD function but on mimicking the effects of its negatively regulated downstream targets. MiR-451a is such a negatively regulated target and was able to increase chemosensitivity by targeting MDR1 in vitro. To date, therapeutics inhibiting MDR1 failed application in medical practice, especially, due to toxicity-associated with pharmacokinetic drug interactions^15^. Here, miR-451a as active substance may circumvent this plight and render treatment more effective. Succeeding research ought to determine how administration of miR-451a may be feasible for future therapy.

## Electronic supplementary material

Supplementary Material and Methods(PDF 129 kb)
