# Supplementary material for: miR-451a abrogates treatment resistance in FLT3-ITD-positive acute myeloid leukemia
Source: Blood Cancer J. 2018 Mar 21;8(3):36. doi: 10.1038/s41408-018-0070-y (PMC5862828; doi:10.1038/s41408-018-0070-y)
Supplement: Supplementary file 1 — Supplementary Material and Methods(PDF 129 kb) [file 41408_2018_70_MOESM1_ESM.pdf]

Supplementary Material and Methods

miRNA expression profiling

MicroRNA expression profiling was performed as previously described[1]. 500ng total RNA extracted using Trizol method was used for small RNA library preparation with the TruSeq™ Small RNA Sample Prep Kit v2 (Illumina) according to the manufacturer's instructions. The barcoded libraries were size restricted between 140 and 165 bp, purified and quantified using the Library Quantification Kit-Illumina/Universal (KAPA Biosystems) according to the manufacturer's instructions. A pool of up to 12 libraries was used for cluster generation per lane. Library DNA at a concentration of 10 pM was clustered using an Illumina cBot according to the SR_Amp_Lin_Block_Hybv8.0 protocol of the manufacturer. Sequencing of 50 bp was performed with an Illumina HighScan-SQ sequencer using version 3 chemistry and the version 3 flow cell according to the instructions of the manufacturer.
For data analysis, Cutadapt software was used to trim adaptor sequences from raw sequences. Alignment to human mature miRNA sequences of miRBase was done from sequences with a length between 15-27 bases with bowtie aligner. Quantification of aligned reads was performed using R/Bioconductor programming environment.

miRNA detection by quantitative real-time PCR

Isolation of RNA, reverse transcription and quantitative PCR reaction of miRNAs was performed as previously described[2]. RNU44 was used for normalization of human microRNAs, snoRNA-135 for normalization of mouse microRNAs. All microRNA primers (miR-451a, sno-RNA135, RNU44) were obtained from Life Technologies.

Flow cytometry and fluorescence activated cell sorting (FACS)

For flow cytometry analysis, $1x10^6$ cells were washed with PBS and stained for 20 min at 4°C with anti-MDR1 antibody (Clone UIC2, e Bioscience) or isotype control. Subsequently, cells were washed in PBS and measured with BD LSR II cytometer using CellQuest software (BD Biosciences). For Annexin V staining, PE Annexin V Apoptosis Detection Kit I (BD Pharmingen) was used according to the manufacturer's instructions. Final evaluation of data was done using NovoExpress 1.0.2 (ACEA Biosciences).

Primary human and murine cell samples

AML patient samples as well as samples from healthy donors were obtained from Leipzig University Hospital (Leipzig, Germany). The study protocols employed were approved by Ethical Committee at the Medical Faculty, Leipzig University and all patients provided informed consent.
Primary human bone marrow mononuclear cells were isolated by means of Ficoll-Hypaque density gradient centrifugation using human Pancol (Pan-Biotech) according to the manufacturer's instructions. Hematopoietic $CD34^+$ cells were isolated from fresh umbilical cord blood using a $CD34^+$ MicroBead kit (Miltenyi Biotec) as previously described [2]. Primary murine samples were separated by the Lineage Depletion Kit (Miltenyi Biotec) according to the manufacturer's instructions.

Cell culture

MV4;11, MV4;11-R and U937 cells were cultured in RPMI 1640 supplemented with 10% FBS and 1% PSG. MV4;11-R cells were treated every two weeks with 140 nm PKC412.. 32D-human FLT3-WT cells were previously described[3] and kept in RPMI 1640 supplemented

with 10% FBS, 1% PSG, 10% WEHI supernatant and 15 mg/ml Blasticidin. 32D-human FLT3-ITD were previously described[3] and kept in RPMI 1640 supplemented with 10% FBS and 1% PSG. Primary umbilical cord blood mononuclear cells were cultured in RPMI 1640 supplemented with 10% FBS, 10 ng/ml hIL-3, 20 ng/ml hIL-6, 100 ng/ml hSCF and 100 ng/ml hFlt-3 ligand. Cytokines were purchased from Immunotools. HEK-293T-ampho and HEK-293TN cells were cultured in DMEM supplemented with 10% FBS and 1% PSG.

Transient transfection and lentiviral transduction

Transfection of MV4;11 cells was performed using Lipofectamine 3000 (Thermo Fisher) according to the manufacturer's instructions. Transfection efficiency was calculated using flow cytometry for GFP and was between 20-40%.

PGhU6 lentiviral vector was kindly provided by Dr. Alberich-Jorda and described previously[4]. Pseudo-viral particles were produced as previously described[4]. MV4;11 and MV4;11-R cells were transduced by spin infection for 90min at 2200 rpm and 35°C with at least 5 MOIs, incubated for 24 hours, washed two times with PBS and sorted for GFP by FACS another 48 hours later.

Cell treatment and viability assessment

Parental and resistant MV4;11 cells were plated at a density of $5*10^5$ cells per 1 mL in 12 well plates. Solvent of therapeutic compounds were always kept at a dilution of 1:1000. After 24 h, fresh media and treatment were added and after 48 h of treatment, cell viability was measured with the MTS cell Proliferation Assay Kit (Abcam #197010) according to the manufacturer's protocol. Probes were analyzed at a wavelength of 450 nm. For the growth curves, cells were plated at a density of 100.000 cells/mL and treated. Cell count was performed daily.

DNA constructs and cloning

Cloning of human precursor miR-451a into pcDNA6.2 and pGhU6 has been done using the appropriate sequence provided by miRBase:
5'-
CTTGGGAATGGCAAGGAAACCGTTACCATTACTGAGTTTAGTAATGGTAATGGTT
CTCTTGCTATACCCAGA-3' and performed as previously described for pGhU6[4] and pcDNA6[3].

For luciferase assay, the 3´UTR of MDR1 amplified from genomic DNA of U937 cells was cloned into the pmiR-GLO vector (Promega). Mutagenenesis was done with the Q5® site directed mutagenesis kit by NEB.

Western Blot

Western blots were performed as previously described[2]. The following antibodies were used: mouse mAb Anti-Mdr-1 (sc-55510, Santa Cruz), rabbit pAb Anti-β-Tubulin (sc-9104, Santa Cruz). Goat-anti-mouse-HRP (sc-2005, Santa Cruz) or goat-anti-rabbit (sc-2004, Santa Cruz) served as secondary antibodies. For chemiluminescence detection, we used Western Blot Ultra or Western Blot Premium Substrate (Licor) and analyzed the membranes using C-Digit Chemiluminescent Western Blot Scanner (Licor). Evaluation of data was performed using ImageJ software (NIH).

Luciferase Assay

MV4;11 cells were co-transfected with pmiR-GLO-MDR1-3'UTR / pmiR-GLO-MDR1-3'UTR mutated and miR-451a mimics / or appropriate control (Dharmacon). As control, MV4;11 cells were co-transfected with pmiR-GLO empty and miR-451a mimics / or

appropriate control (Dharmacon). Firefly- and renilla luciferase activity was measured 24 hours after transfection using Dual-Luciferase Reporter Assay System (Promega) and calculated as ratio between luciferase and renilla activity.

Statistical Analysis

To determine the statistical significance of the results, the student´s t test as well as the Wilcoxon test for patient samples was conducted. A *p*-value of 0.05 or less was considered significant (*), while a *p*-value of 0.01 or less were considered as highly significant (**). The results were depicted as the median ± SD from three independent experiments. All graphs were generated with either Microsoft Excel or Graph Pad Prism 6.

Supplementary references

1. Braun, J., Misiak, D., Busch, B., Krohn, K. & Huttelmaier, S. Rapid identification of regulatory microRNAs by miTRAP (miRNA trapping by RNA in vitro affinity purification). *Nucleic Acids Res* **42**, e66 (2014).
2. Katzerke, C. et al. Transcription factor C/EBPalpha-induced microRNA-30c inactivates Notch1 during granulopoiesis and is downregulated in acute myeloid leukemia. *Blood* **122**, 2433-42 (2013).
3. Gerloff, D. et al. NF-kappaB/STAT5/miR-155 network targets PU.1 in FLT3-ITD-driven acute myeloid leukemia. *Leukemia* **29**, 535-47 (2015).
4. Alberich-Jorda, M. et al. C/EBPgamma deregulation results in differentiation arrest in acute myeloid leukemia. *J Clin Invest* **122**, 4490-504 (2012).
